# Effects of Lordotic Angle of a Cage on Sagittal Alignment and Clinical Outcome in One Level Posterior Lumbar Interbody Fusion with Pedicle Screw Fixation

**DOI:** 10.1155/2015/523728

**Published:** 2015-01-22

**Authors:** Ji-Ho Lee, Dong-Oh Lee, Jae Hyup Lee, Hee Jong Shim

**Affiliations:** ^1^Department of Orthopedic Surgery, College of Medicine, Seoul National University, Seoul 110-744, Republic of Korea; ^2^Department of Orthopedic Surgery, College of Medicine, Seoul National University, SMG-SNU Boramae Medical Center, 20 Boramae-ro 5-gil, Seoul 156-707, Republic of Korea

## Abstract

This study aims to assess the differences in the radiological and clinical results depending on the lordotic angles of the cage in posterior lumbar interbody fusion (PLIF). We reviewed 185 segments which underwent PLIF using two different lordotic angles of 4° and 8° of a polyetheretherketone (PEEK) cage. The segmental lordosis and total lumbar lordosis of the 4° and 8° cage groups were compared preoperatively, as well as on the first postoperative day, 6th and 12th months postoperatively. Clinical assessment was performed using the ODI and the VAS of low back pain. The pre- and immediate postoperative segmental lordosis angles were 12.9° and 12.6° in the 4° group and 12° and 12.0° in the 8° group. Both groups exhibited no significant different segmental lordosis angle and total lumbar lordosis over period and time. However, the total lumbar lordosis significantly increased from six months postoperatively compared with the immediate postoperative day in the 8° group. The ODI and the VAS in both groups had no differences. Cages with different lordotic angles of 4° and 8° showed insignificant results clinically and radiologically in short-level PLIF surgery. Clinical improvements and sagittal alignment recovery were significantly observed in both groups.

## 1. Introduction

The posterior lumbar interbody fusion (PLIF) has gradually shown an increasing trend to restore the structural integrity of intervertebral disc space [1] and maintain stability after decompressing pathogens that compresses the dura or the nerve root in degenerative lumbar diseases inducing instability, radiculopathy, and others. During the initial stage of PLIF, fusion was performed using a tricortical bone graft. However, many studies have reported that successful fusion cases have been verified in the cage and interbody graft by fragmenting the lamina or facet joint bone acquired from intraoperative decompression instead of harvesting additional iliac bone grafts along with the development of the cage [2–4]. Thus, operations implanting cages with only localized autografts obtained during decompression have more frequently been attempted in recent years.

The PLIF could improve discogenic pain by eliminating the disc and increasing the fusion rate more efficiently than posterolateral fusion with tension force by implanting the bone graft into the disc space and operating the compression force in the graft. Moreover, the PLIF is more adequate with regard to load bearing in terms of biomechanical aspects [5] and also maintains lumbar lordosis [5]. A polyetheretherketone (PEEK) cage is more favorable in interpreting postoperative radiographs and more beneficial in fusion evaluation since it is radiolucent compared to metal cages. Moreover, PEEK cages are advantageous in remodeling fusion mass and transferring loads physiologically with less of a stress-shielding effect than metal cages.

An iatrogenic flat back, which develops when lordosis is not recovered after lumbar spine surgery, is known to affect not only the sagittal balance of the whole spine but also the clinical results by negatively affecting the adjacent segment in the long term. Therefore, it is important to recover physiologic lumbar lordosis after surgery because the recovery and maintenance of lumbar lordosis affect load bearing on the surgery site after operation, functions of paraspinal muscles, and energy consumption during gait. Generally, instrumentation using pedicle screws and rods is known to be more effective in lumbar lordosis recovery compared to that using a laminar hook or Luqué ring or no instrumentation at all. Also, PLIF surgery using a cage is known to be more effective in maintaining lordosis compared to that of posterolateral fusion. However, iatrogenic flat back is being reported in PLIF using cages [6]; for this reason, cages of various designs are made and used in surgeries applied with different lordotic angles for optimal lumbar lordosis recovery.

The purpose of this study is to compare the differences of clinical and radiological results including the segmental lordosis and total lumbar lordosis of two different PEEK cages with lordotic angles of 4° and 8°, on the degenerative lumbar disease patients requiring PLIF.

## 2. Materials and Methods

The retrospective, case-control study included patients as subjects who underwent PLIF in the third, fourth, and fifth lumbar spine and the first sacrum spine using PEEK cages with lordotic angles of 4° and 8° (PEEK OIC cages, Stryker, USA), with the pedicle screw and rod system (Xia, Stryker, USA), at the Department of Orthopedic Surgery in our hospital from March 2006 until April 2012. This study was approved by the institutional review board (IRB) at SMG-SNU Boramae Medical Center (16-2012-34). From March 2006 to February 2009, the author performed PLIF consecutively using PEEK cages with lordotic angles of 4°, and from March 2009 to April 2012, PLIF was performed consecutively using PEEK cages with lordotic angles of 8°. Before the PLIF, subjects initially underwent total laminectomy, facetectomy, and discectomy by a single surgeon due to severe spinal stenosis, grade 1 or grade 2 spondylolisthesis, huge herniated intervertebral disc, or other reasons.

This study gained the approval of the institutional review board (IRB) and was conducted retrospectively to assess clinical and radiological differences between the two groups implanted with PEEK cages with lordotic angles of 4° and 8°. Laminar bone and facet bone were fragmented and inserted into the cages (both groups of 4° and 8°) and disc spaces. After the implantation of cages into the disc space, pedicle screws and rods with lordosis were fixed. A custom-made thoracolumbosacral orthosis was worn by all patients in the 4° and 8° cage insertion groups for two months.

### 2.1. Radiological Evaluation

The segmental lordosis and total lumbar lordosis of the vertebral segments inserted with PEEK cages were measured after taking radiographs of standing anteroposterior and standing lateral views of the lumbar spine on the preoperative hospital days, the second postoperative week, the sixth postoperative month, and the first postoperative year. For the segmental lordosis of segments with PEEK cage insertion, the measurement was performed in lordosis forming the upper endplate of the upper vertebra and the lower endplate of the lower vertebra. The S1 vertebra was measured based on the upper endplate of S1. For the total lumbar lordosis, the assessment was done in lordosis comprising the upper endplate of L1 and the upper endplate of S1. We compared the two groups if the cage was inserted parallel to the vertebral endplate and if the anterior, middle, and posterior portions of the inserted cage were all in contact with the endplate in the sagittal plane. Consequently, the differences in segmental lordosis and total lumbar lordosis were examined in the 4° and 8° cage insertion groups over time in each follow-up period.

### 2.2. Clinical Evaluation

The assessment of clinical results was performed by measuring the numeric visual analogue scale (0–10) of low back pain and the Korean Oswestry Disability Index on the preoperative hospital days, the second postoperative week, the sixth postoperative month, and the first postoperative year.

### 2.3. Statistical Analysis

A repeated measures ANOVA was used for analyzing the changes in lordosis over time. A Student's *t*-test was used for the comparison of two groups with different cage angles during the same time period. Furthermore, a repeated measures ANOVA was performed for the analysis of the Oswestry Disability Index and the VAS. Statistical analysis was performed using the IBM SPSS Statistics 2.0. *P* values of less than 0.05 were defined as statistically significant.

## 3. Result

### 3.1. Baseline Characteristics

A total of 160 patients (185 segments) were included in the study. The mean age of the subjects was 64.4 years (±9.9) and 102 subjects were female. The study population comprised 99 patients (115 segments) with 4° and 61 patients (70 segments) with 8°. There were no significant differences in sex, age, operated segments, and preoperative diagnosis between the 4° and 8° groups (Table 1).

### 3.2. Radiological Results

#### 3.2.1. Segmental Lordosis

The proportion of the cage inserted parallel to the vertebral endplate was 96.5% in the 4° group and 96% in the 8° group. There was no significant difference between the two groups.

**Figure 1:**
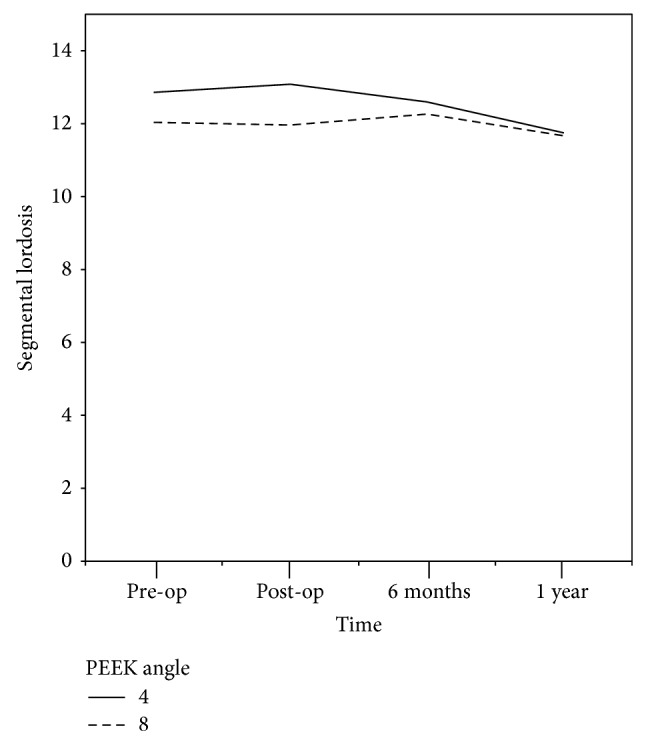
According to segmental lordosis outcome, preoperative segmental lordosis was low in the 8° group. However, the results were insignificant and no differences were found between the two groups by period.

The segmental lordosis angles were 12.9° (±7.6) and 12.6° (±6.7) in the 4° group and 12° (±7.0) and 12.0° (±5.6) in the 8° group during the preoperative days and the first postoperative year, respectively. There were no significant differences detected over time for each follow-up period in both groups (Table 2). Moreover, no significant differences were shown in the outcomes obtained during the same time periods of the second postoperative week and the sixth postoperative month. There was also no significant difference between groups over time (Figure 1).

#### 3.2.2. Total Lumbar Lordosis

**Figure 2:**
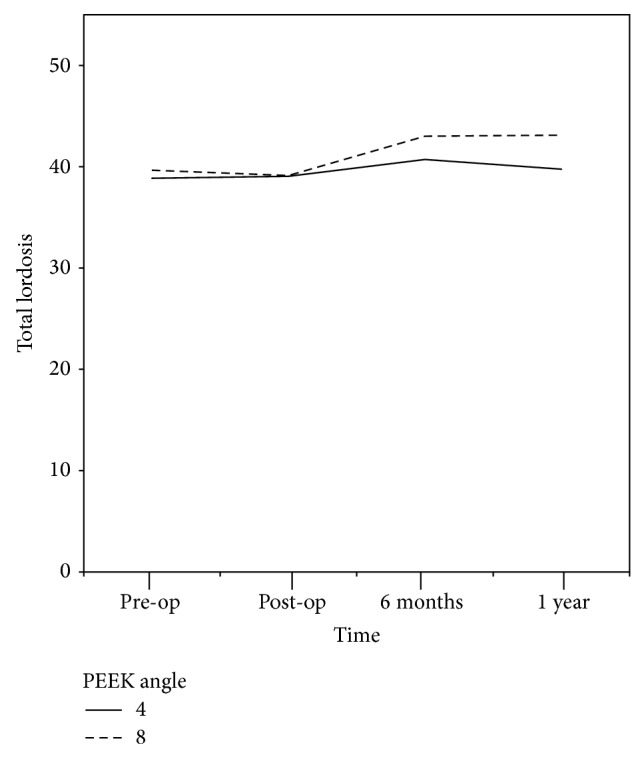
Total lumbar lordosis results. Although the total lumbar lordosis angles were higher in the 8° group than in the 4° group after the first postoperative year, the results were insignificant.

The total lumbar lordosis was 38.9° (±15.2) and 39.6° (±14.5) in the 4° group and 39.7° (±14.5) and 41.0° (±14.0) in the 8° group during the preoperative days and the first postoperative year, respectively (Figure 2). There were no significant differences detected over time by each follow-up period in both groups (Table 2). Moreover, no significant differences were shown in the total lumbar lordosis of each group during the same time periods of the preoperative days, the second postoperative week, the sixth postoperative month, or the first postoperative year. There was no significant difference in each period in the 4° group. However, significant differences were shown in the 8° group over time (*P* value = 0.005). Although no significant differences were found between the preoperative and postoperative lordosis angles, the total lumbar lordosis exhibited a significant increase after the first six postoperative months compared with the immediate postoperative days (*P* value = 0.002). No significant changes were observed in the total lumbar lordosis after the sixth postoperative month or the first postoperative year.

### 3.3. Clinical Results

#### 3.3.1. Oswestry Disability Index

**Figure 3:**
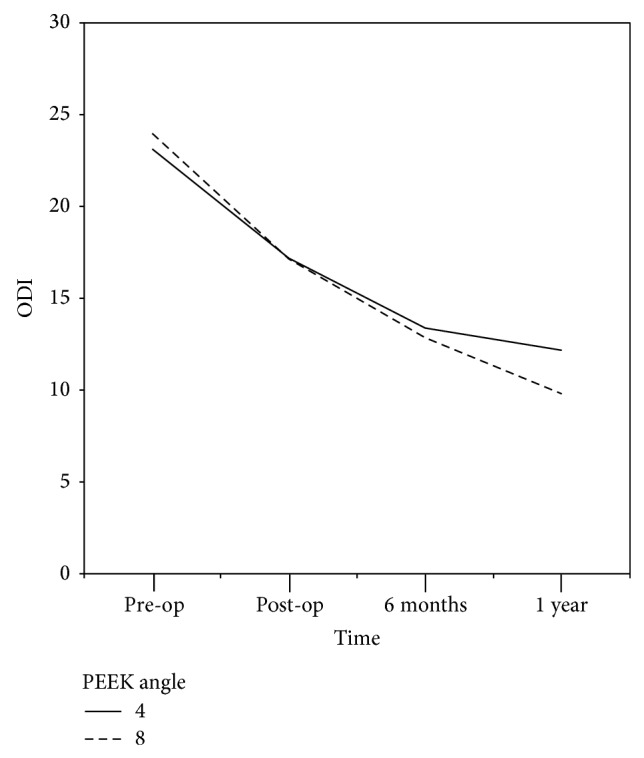
Oswestry Disability Index results. Although the ODI scores were lower in the 8° group than in the 4° group after the first postoperative year, there were no significant differences between the two groups over time.

The Oswestry Disability Index scores were 23.1 (±5.5) and 18.3 (±6.3) in the 4° group and 24 (±5.4) and 18.4 (±7.1) in the 8° group during the preoperative days and the first postoperative year, respectively (Figure 3). There were no significant differences found in each period between the two groups (Table 3). In addition, there were no differences in the 4° and 8° groups with the significance probability of less than 5% during the same time period. However, the ODI scores were significantly improved after the second postoperative week, the sixth postoperative month, and the first postoperative year in the 4° group compared with the states before surgery (*P* < 0.001). The ODI scores were also significantly improved after the second postoperative week, the sixth postoperative month, and the first postoperative year in the 8° group (*P* = 0.003).

#### 3.3.2. Visual Analogue Scale of Low Back Pain

**Figure 4:**
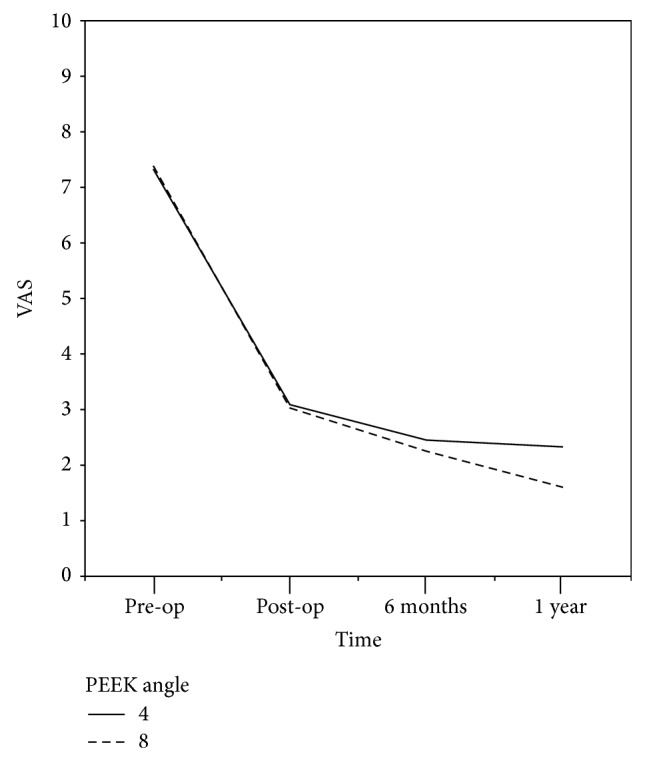
Visual analog scale of low back pain results. The VAS scores were lower in the 8° group than in the 4° group after the first postoperative year, showing no significant differences. However, the VAS values were significantly improved in both groups by each period compared with the preoperative VAS scores.

The visual analogue scales of lower back pain were 7.3 (±1.2) and 2.3 (±1.3) in the 4° group and 7.4 (±1.7) and 1.6 (±1.6) in the 8° group during the preoperative days and the first postoperative year, respectively (Figure 4). There were no significant differences found during each period between the two groups (Table 3). In addition, there were no differences in the VAS values of the two groups with the significance probability of less than 5% during the same time period. However, the VAS values were significantly improved after the second postoperative week, the sixth postoperative month, and the first postoperative year in the 4° group compared to the states before surgery (*P* < 0.001). The VAS values were also significantly improved after the second postoperative week, the sixth postoperative month, and the first postoperative year in the 8° group (*P* = 0.009).

## 4. Discussion

Physiological sagittal alignment plays a pivotal role in the body balance and pain of spinal disorders. Furthermore, the preservation of sagittal alignment is crucial in spinal reconstructive surgery, since it also affects potential complications. Therefore, iatrogenic flat back should be avoided during surgery [7, 8]. Along with a recent increase in the incidence of lumbar fusion surgery, the importance of lumbar lordosis maintenance has been highlighted [9, 10]. The PLIF is a standard surgical treatment method for the recovery of disc height, stabilization of the unstable degenerated intervertebral disc area, load transfer of the anterior structure, the recovery of segmental alignment, and successful fusion acquisition using interbody cages. In particular, the recovery of total and segmental lordosis needs to be watched more carefully during PLIF surgery [11].

The use of cages has been advanced after clinically applying cylindrical and rectangular-shaped cages in the lumbar region of the spine since the 1990s [12–14]. Cages have been extensively used in intervertebral fusion surgery of the cervical and lumbar spines. Various custom-made cages with different lengths, widths, and angles could be appropriately used depending on the patient, the level of spine, and other individual circumstances. The most commonly used cages are classified into threaded and impacted cages based on the design. Although cages are made of similar materials, such as titanium, carbon, and PEEK, there could be differences in contact areas among end plates depending on various designs [15]. The study was able to clinically confirm significant alleviation in all cage insertion groups with the lordotic angles of 4° and 8° compared to the clinical conditions before surgery. Moreover, patients recovered to the preoperative states of total lumbar lordosis and segmental lordosis. However, no significant differences were shown in lumbar segmental lordosis between the two groups. The average segmental lumbar lordosis angles were approximately 13°, 20°, and 28° in L3-4, L4-5, and L5-S1, respectively. The maximum lordotic angle was shown in L5-S1, in particular [16]. Diedrich et al. [17] reported that there were no significant differences in the recovery of lumbar lordotic angle with the use of rectangular cages without lordotic angle and the use of a wedged cage with a 4° lordotic angle. In addition, they were unable to obtain statistically significant results due to an insignificant difference in the angle of 4°.

There were also no significant differences in the segmental lordosis (*P* = 0.943) and total lumbar lordosis (*P* = 0.263) depending on lordotic angles of 4° and 8° in this study. If the cages are not inserted parallel to the vertebral endplate or subsidence occurred asymmetrically, the segmental lordosis or the total lumbar lordosis, caused by the cage, might be distorted regardless of the lordotic angles of the cage. However, the proportion of the cages inserted parallel to the vertebral endplate is over 95% in both groups. Therefore, the angle difference of 4° cage had no significant influence on the segmental lordosis in one level, and one level PLIF was not influenced by the wrong position of the cages.

This could be attributable to the lordotic angles of cages where cage shapes make incomplete contact with the vertebral endplate due to an anatomically concave structure. Since the original segmental lordotic angle of lumbar spine is over 10° [18, 19], all cages with angles of 4° and 8° are not sufficient to have a significant impact in anatomical lordotic angles [20]. Similar to the results of some studies, different cage designs such as horizontal cylinder type and open box type do not influence sagittal alignment during the PLIF [21].

On the other hand, lordosis was formed through the procedures of rod bending and screw compression, among others, during the instrumentation of the pedicle screw and rod system, unlike a single insertion of a cage. The lordosis formed during the process could be more influential than just the lordotic angles of cages. There were some limitations to maintain satisfying lumbar lordosis only with the application of cage geometry [22].

However, the investigation was confined to the one level PLIF. In addition, we could not assume that the same results might have been acquired from the multilevel PLIF as well, since the study included patients within the normal range of preoperative sagittal alignment. Other outcomes are anticipated to be obtained in multilevel surgery, preoperative lumbar kyphosis, and others.

According to the results of our study, the Oswestry Disability Index and the VAS of low back pain clinically showed significant improvement in both groups after surgery. However, there was no difference between the two groups and the alterations in angles did not generate any clinical differences. The reasons are thought to be attributable to insignificant differences between the two groups in the level of decompression, fusion rate, and sagittal alignment affecting clinical results. The study results aligned with the outcomes of previous studies [11, 22–24] where the use of pedicle screws and cages, technical alterations including approach methods, PLIF, posterolateral fusion, transforaminal lumbar interbody fusion, and others resulted in no significant differences in clinical results such as low back pain.

## 5. Conclusion

In conclusion, cages with different lordotic angles of 4° and 8° showed insignificant results clinically and radiologically in short-level PLIF surgery. Clinical improvements and sagittal alignment recovery were significantly observed in both groups. Besides the lordotic angle of a cage, securing lordosis needs to be taken into account by using intraoperative rod bending, screw compression, and other techniques for the improvement of sagittal alignment.

## Figures and Tables

**Table 1:** Demographic data. No significant differences were shown in age, sex, surgical segments, and preoperative diagnosis between the 4° and 8° groups.

Demographic variables	PEEK 4°	PEEK 8°	*P* value
Number of patients	99/160	61/160	NA
Application	115/185	70/185	NA
Age distribution (years)	64.3 ± 10.4	64.5 ± 9.0	0.896^*^
Sex (M : F)	35 : 64	23 : 38	0.866^*^
L3-4	25/115	10/70	NA
L4-5	76/115	49/70	NA
L5-S1	14/115	11/70	NA
Listhesis : stenosis : HIVD	34 : 75 : 6	23 : 42 : 5	NA

^*^By Student's *t*-test.

NA: not applicable.

**Table 2:** Segmental lordosis and total lumbar lordosis of both groups.

	Pre-op segmental lordosis	2 wk segmental lordosis	6-month segmental lordosis	1-year segmental lordosis	Pre-op total lordosis	2 wk total lordosis	6-month total lordosis	1-year total lordosis
PEEK (4)	12.9 (±7.6)	13.1 (±6.4)	12.6 (±6.5)	12.6 (±6.7)	38.9 (±15.2)	39.1 (±13.1)	40.7 (±14.7)	39.6 (±14.5)
PEEK (8)	12.0 (±7)	12 (±6.2)	12.3 (±6.5)	12.0 (±5.6)	39.7 (±14.5)	39.1 (±12.9)	43.0 (±13.7)	41.0 (±14.0)
*P* value^*^	0.493	0.744	0.278	0.943	0.973	0.754	0.330	0.263

^*^By *t*-test.

**Table 3:** Oswestry disability index and visual analogue scale of low back pain of both groups.

	Pre-op Oswestry	2 wk Oswestry	6-month Oswestry	1-year Oswestry	Pre-op VAS	2 wk VAS	6-month VAS	1-year VAS
PEEK (4)	23.1 (±5.5)	17.2 (±4.1)	13.4 (±4.0)	18.3 (±6.3)	7.3 (±1.2)	3.1 (±1.5)	2.5 (±1.5)	2.3 (±1.3)
PEEK (8)	24 (±5.4)	17.1 (±5.4)	12.9 (±4.2)	18.4 (±7.1)	7.4 (±1.7)	3.0 (±1.7)	2.3 (±1.3)	1.6 (±1.6)
*P* value^*^	0.399	0.959	0.633	0.084	0.844	0.889	0.612	0.150

^*^By *t*-test.
